# Hypokalemic periodic paralysis as the first sign of thyrotoxicosis- a rare case report from Somalia

**DOI:** 10.1186/s13044-023-00158-4

**Published:** 2023-06-12

**Authors:** Hawa Nuradin Mohamed, Abdi Karim Ahmed Ghedi, Sevgi Ozturk, Mohamed Osman Omar Jeele, Ahmed Muhammad Bashir

**Affiliations:** Department of Internal Medicine, Mogadishu Somali Turkey Training and Research Hospital, Mogadishu, Somalia

**Keywords:** Thyrotoxicosis, Periodic paralysis, Hypokalemia, Somalia

## Abstract

**Background:**

Thyrotoxic hypokalemic periodic paralysis (THPP) is a rare complication of hyperthyroidism characterized by thyrotoxicosis, hypokalemia, and paralysis. It is the most common form of acquired periodic paralysis. THPP is precipitated by strenuous exercise, a high carbohydrate diet, stress, infection, alcohol, albuterol, and corticosteroid therapy. It is most common in Asian men with hyperthyroidism and exceptionally rare in black people.

**Case Presentation:**

A 29-year-old man was admitted to the emergency department in Somalia with a sudden onset of paralysis after a high carbohydrate meal. Laboratory investigations showed low serum potassium 1.8 mEq/l (3.5–4.5), and biochemical thyrotoxicosis with TSH 0.006 miu/l (0.35–5.1), total T3 3.2 ng/ml (0.9–2.8) and total T4 13.5 ng/ml (0.6–1.2). He was successfully treated with potassium chloride infusion and an antithyroid drug, methimazole.

**Conclusion:**

To prevent life-threatening cardiac and respiratory complications, it is critical to consider and diagnose THPP early, even in populations where the condition is rare.

## Introduction

Thyrotoxic hypokalemic periodic paralysis (THPP) is a rare endocrine disorder characterized by the triad of thyrotoxicosis, hypokalemia, and sudden muscle weakness. It is most commonly seen at night [1]. The proximal muscles are more severely affected than the distal ones. The lower limbs are usually affected first, followed by the girdle muscles and the upper limbs. The sensory system remains unaffected [2].

THPP is a rare cause of paralysis in the general population. It is most common in the Asian populations, where it affects about 2% of hyperthyroid individuals. In contrast, it is rare in non-Asian populations, where the incidence is estimated to be between 0.1 and 0.2% among patients with hyperthyroidism [3]. It occurs 70 times more frequently in men than in women and typically occurs between the ages of 20 and 40 [4].

The mechanism through which hyperthyroidism results in hypokalemic periodic paralysis is unknown. Thyroid hormone improves tissue response to beta-adrenergic stimulation, which increases sodium-potassium ATPase activity on the skeletal muscle membrane in combination with thyroid hormone [5].

The presence of both profound hypokalemia and biochemical thyrotoxicosis (increased serum levels of thyroxine and triiodothyronine with suppressed TSH) confirms the diagnosis of THPP. Its treatment comprises of correcting hypokalemia for immediate reversal of paralysis and restoring normal thyroid status to prevent future THPP attacks [6].

Here, we report a case of a young man, who presented to the emergency department in Somalia with a sudden onset of paralysis due to THPP.

## Case presentation

A 29 years old man was admitted to the emergency department with a sudden onset of paralysis. He had gone to bed and slept at 15:30 with no symptoms and awoke in the evening unable to move his upper or lower extremities. The weakness was bilateral, and also involved proximal muscles of shoulders and hips. He had no dysphagia and shortness of breath. He could talk and move his neck. Before he went to the bed, he had eaten a high carbohydrate meal. He denied fever, recent diarrhea, chest pain, and a history of strenuous exercise. Before this episode, the patient was experiencing bilateral cramp-like leg pain for 1 month, but he had not sought medical advice. He had no past history of any chronic disease, or a family history of such episodes.

On examination, the patient was conscious, alert, and oriented to time, place, and person. His blood pressure was 120/60 mmHg, heart rate of 75 beats per minute, the temperature of 36.5 degrees centigrade, respiratory rate 36 breaths per minute, and sp02 96% in room air. He had no jaundice, conjunctival hemorrhage, photophobia, palpable mass on his neck or lymphadenopathy. There was no lower limb edema. The lung and abdominal examinations were unremarkable. Neurological examination showed reduced muscle power in all four limbs to 1/5, his cranial nerves were intact, and there was no bladder or bowel dysfunction.

**Table 1:** Laboratory findings at presentation

Tests	Results	Reference Range
Blood pH	7.39	7.35–7.45
Bicarbonate	20.3 mmol/L	22–32
pCO2	32 mmHg	35–45
White blood count	5.69 × 1000/mm^3^	4–10
Hemoglobin	14.2 g/dL	13–17
Platelet count	180 × 1000/mm3	100–430
Glucose	155 mg/dL	60–200
Creatinine	0.52 mg/dL	0.6–1.35
Urea	17 mg/dL	10–45
Aspartate aminotransferase	31 U/L	0–35
Alanine aminotransferase	33 U/L	0–45
Total Protein	6.9 g/dL	6.4–8.3
Calcium	9.4 mg/dL	8.3–10.6
Magnesium	2.3 mg/dL	1.87–2.5
Sodium	140 mEq/L	135–145
Potassium	1.8 mEq/L	3.5–4.5
Chloride	104 mEq/L	95–106
C-reactive protein	0 mg/L	0–10
TSH	0.006 miu/L	0.35–5.1
Total T3	3.2 ng/mL	0.9–2.8
Total T4	13.5 ng/mL	0.6–1.2
Anti-thyroid peroxidase (TPO) antibodies	0.64 iu/mL	< 116
Hepatitis B surface antigen (HBsAg)	Negative	
Hepatitis C antibody (Anti-HCV)	Negative	
HIV antibody (Anti-HIV)	Negative	

His initial investigations showed marked hypokalemia at 1.8 mEq/L (reference range 3.5–4.5) **(**Table 1**)**. Electrocardiography showed T wave inversion, ST depression and prominent U waves.

We started an intravenous infusion of potassium chloride at 10 mEq/L/hr. After 6 h of potassium replacement, he regained his distal muscle movement when his serum potassium was 2.4 mEq/L. His neurological symptoms completely resolved after 12 h of potassium supplementation, when his potassium was 4.24 mEq/L.

To determine the cause of his hypokalaemic paralysis, we checked his thyroid function tests, which showed biochemical thyrotoxicosis with fully suppressed thyroid-stimulating hormone (TSH) with elevated total T3 and total T4 levels **(**Table 1**)**. His anti-thyroid peroxidase (TPO) antibodies were negative, TSH-receptor antibodies were not checked. Thyroid ultrasound revealed increased thyroid size, with hypervascular parenchymal echogenicity.

The next day, the patient was stable, and we discharged him with methimazole 30 mg/day, with a follow-up in the outpatient clinic.

## Discussion

Our patient exhibited lower motor neuron quadriparesis with no bladder or bowel problems in association with severe hypokalemia and biochemical thyrotoxicosis, consistent with the diagnosis of THPP. Flaccid quadriparesis can also be caused by Guillain-Barre Syndrome, transverse myelitis, myasthenia gravis, tick paralysis, or botulism. There was no sensory impairment or bladder/bowel involvement in our patient, therefore transverse myelitis was ruled out. Typical clinical features of myasthenia gravis such as diurnal fluctuation of weakness, facial weakness, or eyelid drooping were absent. There were no symptoms of botulism like fever or food poisoning. The patient was first suspected of having Guillain-Barre syndrome, but an arterial blood gas (ABG) showed hypokalemia with a normal pH. The patient denied any personal or family history of such occurrence of paralysis. Familial periodic paralysis, spontaneous periodic paralysis, and THPP are other differential diagnoses in our patient. The diagnosis of THPP was confirmed following thyroid function test showing biochemical thyrotoxicosis in association with severe hypokalaemia and paralysis.

Hypokalemic periodic paralysis is a rare and can be either primary (familial) or secondary (environmental). Hyperthyroidism and a variety of other disorders, such as hyperaldosteronism, diabetic ketoacidosis, nephrotic syndrome, medications, acute tubular necrosis, laxative or diuretic usage, diarrhea, and vomiting, are examples of secondary causes [7]. To exclude the primary causes (familial periodic paralysis), we couldn’t do a genetic test as it is not available in our country. THPP is common in Asian men, including Chinese, Japanese, Vietnamese, Filipinos, and Koreans, and Graves’ disease is the most common cause of hyperthyroidism associated with THPP [8]. THPP is an uncommon condition, so additional diagnoses such as familial hypokalemic periodic paralysis, myasthenia gravis, Guillain-Barre Syndrome, viral and inflammatory myopathies, transverse myelitis, cord compression, and other electrolyte abnormalities should be evaluated. These causes can be ruled out based on the history, physical examination, and other tests [9]. Many patients with THPP have no overt symptoms or signs of thyrotoxicosis. Because the condition is uncommon in non-Asian populations, it is commonly misdiagnosed [2].

A high-carbohydrate, high-salt diet, alcohol consumption, trauma, menstrual cycle, infections (including viral gastroenteritis), certain medications (e.g., steroids, diuretics, epinephrine, acetazolamide, and insulin), and strenuous exercise are potential precipitating factors for THPP in patients with underlying thyrotoxicosis [10]. Our patient had a high carbohydrate meal before he slept.

Deaths from respiratory paralysis and heart failure have been reported in THPP, even though the condition is rarely life-threatening and rarely involves the cranial nerves. The paralysis usually resolves in 3–36 h, in the reverse sequence in which it appeared. Serious morbidity, such as dysrhythmias, ventilator failure, and death, is uncommon [4].

The pathophysiology of THPP remains unclear. Membrane excitability and muscle contraction are controlled by sodium, chloride, calcium, and potassium channels on cell membranes. Disruption of any of these cellular transport mechanisms, particularly the potassium ion channel, can result in aberrant muscle contractility and paralysis. The main defect in THPP is a rapid increase in intracellular potassium. This is related to genetic abnormalities in the Na+/K + ATPase pump in the majority of instances [4]. Thyroid hormone stimulates Na+–K + ATPase in skeletal muscle via genomic mechanisms that work on thyroid hormone-responsive regions to upregulate the transcription of the Na+–K + ATPase gene, as well as nongenomic mechanisms that raise the pump’s intrinsic activity or facilitate membrane insertion. Hyperthyroidism may enhance the activation of pump-action by b2-adrenergic agonists by increasing intracellular cAMP synthesis. Hyperinsulinemia is also seen in acute THPP attacks, and insulin release in response to oral glucose challenge is increased in patients with THPP, implying that insulin plays a role in the pathophysiology of hypokalemia in THPP [1].

Aside from potassium supplements, acute THPP is treated by immediately reducing thyroid hormone levels. It is vital to remember that during the recovery period, the release of potassium and phosphate from the cells can contribute to rebound hyperkalemia. Intravenous potassium therapy for hypokalemia causes a faster response than oral supplements. Prophylactic potassium dosage between attacks has not been proven to be useful. Non-specific beta-blockers have been shown to reduce the frequency and severity of episodes. Hyperthyroidism should be controlled to prevent attacks of muscle weakness [10]. Only a few cases of THPP have been reported from African populations [11].

## Conclusion

THPP as the first manifestation of thyrotoxicosis is rare, as our patient had no history of hyperthyroidism. This disorder is rare among African populations. THPP is frequently misdiagnosed in non-Asian populations because it resembles familial periodic paralysis. Early detection, effective treatment, and prevention of rebound hyperkalemia will all benefit from an increased awareness of this disorder.

## Data Availability

The datasets used and/or analysed during the current study are available from the corresponding author on reasonable request.

## Abbreviations

THPP: Thyrotoxic hypokalemic periodic paralysis
TSH: Thyroid stimulating hormone
ATP: Adenosine triphosphate
cAMP: Cyclic adenosine monophosphate
